# Computational evaluation of an extra-aortic elastic-wrap applied to simulated aging anisotropic human aorta models

**DOI:** 10.1038/s41598-019-56609-2

**Published:** 2019-12-27

**Authors:** Christian Legerer, Zakaria A. Almsherqi, Socrates Dokos, Craig S. McLachlan

**Affiliations:** 1 Rural Clinical School, Faculty of Medicine, University of New South Wales, Sydney, NSW 2052 Australia; 2 Yong Loo Lin School of Medicine, National University of Singapore, Singapore, 119228 Singapore; 3 Graduate School of Biomedical Engineering, Faculty of Engineering, University of New South Wales, Sydney, NSW 2052 Australia; 4 Faculty of Health, Centre for Healthy Aging, Torrens University, Sydney, NSW 2009 Australia

**Keywords:** Cardiac device therapy, Computational science

## Abstract

Structural changes occurring to the aortic wall can result in vascular stiffening. This is represented by a loss of vascular compliance during pulsatile flow, resulting in increased systolic and pulse blood pressure, particularly in populations aged 50 and over. Aortic stiffness is thought to be permanent and an active de-stiffening strategy is yet to be developed. Extra aortic elastic wrapping has been proposed as a surgical technique to boost aortic distensibility and treat hypertension in the elderly. Previously, *in-vivo* and *in-vitro* testing have suggested a pulse-pressure reduction potential of elastic wrapping in the stiffened aortas. Herein, we explore the feasibility of elastic aortic wrapping to improve simulated aortic compliance across the age span. Detailed computational studies of the anisotropic aortic wall mechanics, using data from human subjects, were performed, evaluating key performance properties for the interaction between the aortic wall and elastic aortic wrap procedure. Main determinants of the procedure’s efficiency are identified using a pre-defined aortic stiffness and wrap elasticity. Finite element analysis predicts that segmental aortic distensibility can be increased if elastic wrapping is applied to a simulated stiff aorta. Elastic aortic wrapping is calculated to have little impact on the compliance of an initially distensible aorta.

## Introduction

Age-related stiffening of the aorta is a significant cause of isolated systolic hypertension in the population aged 50 and above^1,2^. Large conduit arteries, such as the aorta, stiffen due to structural changes occurring in the arterial wall. As a result, vascular compliance during pulsatile flow is reduced, producing an elevated systolic but not diastolic pressure.

Over the course of a human life span, repetitive expansion of the aortic wall causes elastin fibers within the media to rupture and fragment, causing the aorta to lose its ability to distend. Observed is a reduction in distensibility^3^. Other processes, such as accumulation of arterial calcium and collagen, also contribute to the aorta’s reduction in distensibility^4^. An increased aortic stiffness leads to a higher primary pressure wave amplitude and velocity, which also accelerates the secondary reflected arterial pressure waves from the periphery^5^. At present, permanent aortic stiffening is not primary targeted by medical treatment^6^. Hence an active de-stiffening strategy of the aging or diseased aortic wall is required.

Iliopoulos^7^ and Gillies *et al*.^8^ first investigated elastic aortic wrapping as non-pharmacological approach to reduce hypertension by targeting the aortic wall. An elastically externally wrapped stiff aorta was demonstrated to distend more during systolic pressure rise (during normal pulsatile flow), increasing local distensibility. Advantages of the procedure are suggested to be the following: (1) Reduction in systolic and pulse pressure, (2) Increase in diastolic pressure, (3) Reduced cardiac afterload and ventricular oxygen demand, (4) Improved coronary perfusion, and (5) Decline in micro-vascular and cerebrovascular events^7,8^. It is hypothesized that this is achieved by dampening of the primary pressure wave and delaying the returning secondary wave. This concept of age-related aortic stiffness affecting hemodynamics is supported by multi-branched mathematical models that have also indicated a reduced aortic impedance and pulse pressure^9^. A maximal pulse pressure reduction of 23% has been reported if compliance is increased. On the contrary, elastic aortic wrapping of a compliant descending thoracic aorta in a sheep was shown to reduce distensibility^7^. Elastic wrapping should be distinguished from other surgical procedures such as the PEARS procedure, where the aorta is wrapped with a relatively non-elastic material, commonly Dacron^10^. To date elastic aortic wrapping has not been tested in humans.

In recent decades, translational research has used numerical investigations to understand vascular mechanics and hemodynamics. Computer simulations can accurately predict *in-vivo* vascular behavior^11,12^. In this study we have implemented a three-dimensional computational model based on anisotropic aortic wall properties of human subjects, considering elastin and collagen material responses, to quantify distensibility improvements associated with elastic wrapping of the stiffened ascending aorta. Two aortic models that simulated stiffnesses of ‘middle age’ (distensibility 6 [10^−3^ mmHg^−1^]) and ‘elderly’ population (distensibility 1 [10^−3^ mmHg^−1^]) were selected to study distensibility changes of elastically wrapped aortic segments. Maximal compression pressures and radii are also assessed. Moreover, we discuss how elastic wrapping may alter the aortic pressure wave if applied to an aging and stiffened simulated human aorta.

## Results

We implemented a sequential set of simulations, firstly modelling elastic wrapping of a stiffened human aorta (distensibility 1 [10^−3^ mmHg^−1^]), followed by a variation of the compression pressure imposed on the aortic wall and finally comparing these findings with a more compliant aorta (distensibility 6 [10^−3^ mmHg^−1^]). Physiological inflation pressures of 120/80 mmHg (systolic/diastolic) and 150/80 mmHg were applied to two aortic wall models, ‘middle aged’ and ‘elderly’ respectively. Aortic displacement is reported for an elastically wrapped section and compared with untreated segments proximal and distal to the elastic wrap. An aorta model representing the stiffened wall of an aging human subject (‘elderly’) associated with isolated systolic hypertension was used to demonstrate the feasibility of elastic wrapping to improve aortic compliance. We next proceeded to demonstate that decreasing elasticity of the wrapping material would decrease compliance. Additionally, we compared these findings with a more compliant aortic wall (‘middle aged’), to demonstrate the relation between aortic distensibility and the feasability of elastic wrapping, on further improving aortic compliance.

The cross-sectional definition of aortic distensibility is: AD = (maximum area - minimum area)/(minimum area x pulse pressure) in [10^−3^ mmHg^−1^]. Note that throughout this study the luminal cross-sectional area was used to calculate distensibility. Aortic distensibility is approximately 9 [10^−3^ mmHg^−1^] in children^13^ and can decrease to 0.7 [10^−3^ mmHg^−1^] in elderly patients with hypertensive target organ damage^14^. In untreated sections proximal and distal to the elastic wrap, our aortic models exhibited distensibilities of 6 [10^−3^ mmHg^−1^] (‘middle aged’) and 1 [10^−3^ mmHg^−1^] (‘elderly’), which are typical for a population older than 40 and 70 years, respectively^15^.

### Wall displacement of the stiffend aorta

**Figure 1:**
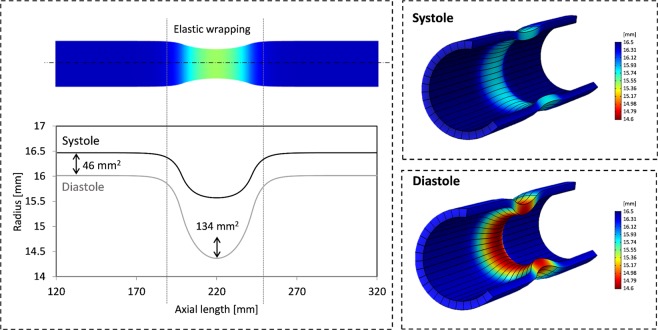
Left: Longitudinal view of wrapped aortic segment and untreated reference for an aortic stiffness model representing the elderly (AD = 1 [10^−3^ mmHg^−1^]). Inner radial wall displacement along axial model length for elastic wrap compression of 10000 Pa. Cross-sectional increase during systolic expansion for untreated and wrapped segments are annotated. Right: Contour plots of radial displacement along ascending aorta model are displayed through sectional cuts. Note, illustrations are scaled for better visualization. Importantly, expansion of wrapped segments greatly exceed untreated model ends.

Figure 1 illustrates our simple model of an elastically constricted aortic segment. Inner aortic radius is plotted versus axial length. The 3D cut sections illustrate aortic wall movement at systolic and diastolic pressures of hypertensive subjects (150/80 mmHg, respectively). Stiffness properties that simulate the elderly patient’s aorta were applied. The outer vessel wall was compressed by a pressure of 10000 Pa (75 mmHg) in diastole and held constant during systole. This resulted in a radial constriction of 11.6% in reference to diastolic dimensions. Luminal cross-sectional expansions for untreated and wrapped sections highlight the improved distensibility of a treated section (Fig. 1). Proximal and distal to an elastically wrapped segment, our model exhibited small radial distentions (0.44 mm) upon a systolic pressure rise of 70 mmHg. Specifically, we demonstrate that a central elastically-wrapped section has the ability to expand substantially further in systole (up to 1.4 mm), which increases aortic luminal cross section by 134 mm^2^, compared to 46 mm^2^ if left untreated. This is equivalent to a local increase in aortic distensibility from an untreated 1 [10^−3^ mmHg^−1^] to 3 [10^−3^ mmHg^−1^] in an elastically wrapped section. We believe that constant compression forces during systole are an assumption, which may be difficult to achieve practically. Therefore, we investigated the role of rising compression forces during systole, reflecting wrap material elasticity.

### Additional compression forces

**Figure 2:**
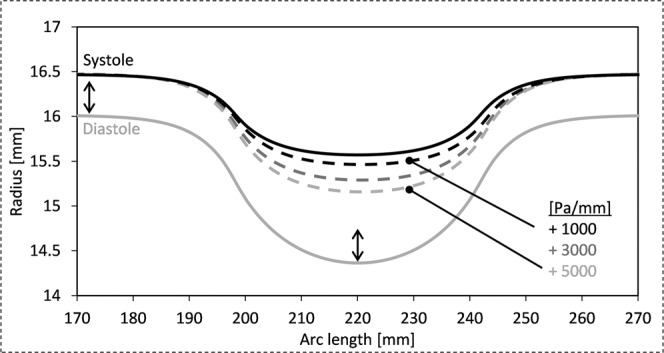
Radial displacement of luminal wall for diastolic and systolic pressures. Wrapped segment was subjected to 10000 Pa diastolic compression, increasing during systole with 1000, 3000 and 5000 Pa/mm. Distensibility improvement dependent on the initial compression force and wrap elasticity. Aortic model with material properties of elderly patient (AD = 1 [10^−3^ mmHg^−1^]).

Specifically, Fig. 1 presents the simulation results of an elastic material wrapped around the aortic model to exert 10000 Pa of evenly distributed compression force which was held constant in systole. However, the systolic expansion of the aorta will cause the elastic wrap to stretch, which will effectively increase the compression pressure. Assuming a linear elastic wrapping material, the rising compression pressure is determined by the Young’s modulus of the wrapping material. We applied an additional 1000 to 5000 Pa per millimeter expansion to the outside of the aortic wall. Figure 2 illustrates the decreasing distention of the wrapped segment in relation to the additional compression forces, 1000, 3000 and 5000 Pa/mm. The grey solid line represents diastolic wall displacement for all compression cases. A maximally achievable increase in luminal cross section during systole of 134 mm^2^ for the ‘elderly’ stiffness model was reduced to 94 mm^2^ for an additional compression force of 5000 Pa/mm (equivalent to a Young’s modulus of 75 kPa). Nevertheless, the distensibility of a wrapped aorta compared to an untreated segment of the simulated aorta was significantly improved, despite an increasing force necessary to expand the elastic wrap material. Having demonstrated the efficacy of elastic wrapping with constant and increasing compression forces in the stiff aorta, we next wished to compare these results with a more compliant aortic wall model.

### Wall displacement in different stiffness models

**Figure 3:**
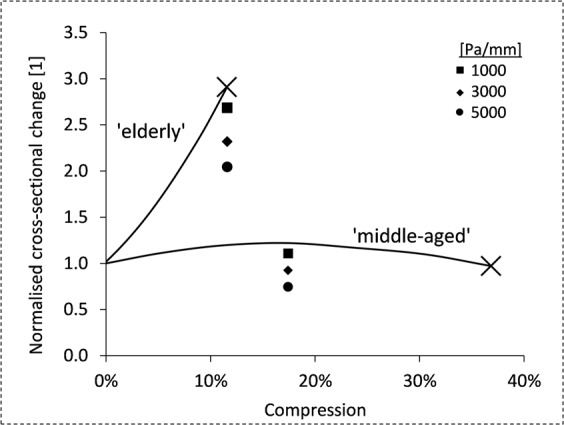
Normalised luminal cross-section increases during systole plotted versus radial compression as a result of elastic wrapping. Stiffness models ‘middle aged’ (AD = 6 [10^−3^ mmHg^−1^]) and ‘elderly’ population (AD = 1 [10^−3^ mmHg^−1^]) are contrasted. Results for various Young’s moduli of wrapping materials are indicated (1000, 3000 and 5000 Pa/mm); Normalization with reference to 0% compression values.

Figure 3 contrasts simulation results of elastic wrapping for two aortic models representing age-specific stiffness of the ‘middle aged’ and ‘elderly’ population. The ordinate reflects changes in aortic luminal cross-section from diastole to systole, which is presented as a function of radial constriction exerted by elastic wrapping in diastole. Percentage compression depended on the compression force applied on the outer aortic wall. Point markers indicate reduced expansion due to additional compression forces. Common scales for normalization were 0% compression values. X markers indicate the maximal constriction without shape deformation of the aortic wall (37% and 10% reduction in radius relative to the diastolic dimensions). Note that compression forces higher than 12000 Pa applied to the ‘elderly’ stiffness model lead to buckling or shape deformation of the aortic model. Our ‘middle aged’ aortic stiffness model showed creasing of the aortic wall at compression forces greater than 14000 Pa. As discussed in our previous study^16^, increasing compression forces beyond a stiffness-dependent threshold can result in luminal creasing.

**Figure 4:**
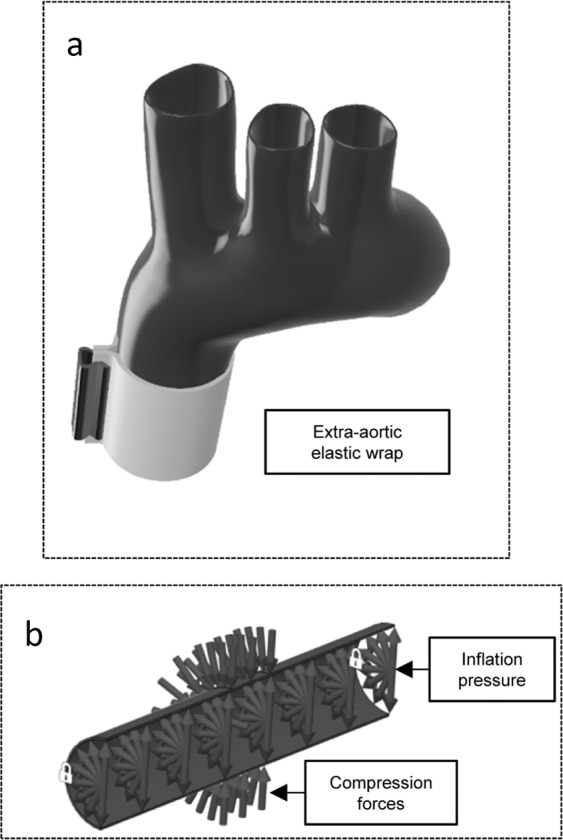
(**a**) Schematic of elastic aortic wrapping procedure; (**b**) Boundary conditions of aortic model section: Inflation pressure and compression force (dimensions of the model aorta geometry are reduced for the purpose of illustration); Illustrations prepared with Autodesk Fusion 360 (California, U.S.).

Our stiffness models ‘middle aged’ and ‘elderly’ predict luminal cross-sectional increases of 145 mm^2^ and 46 mm^2^, respectively, for untreated sections to the left and right of the elastic wrap when inflated at a pressure of 120/80 and 150/80 [mmHg] systolic/diastolic. Untreated reference aortic distensibilities of 6 and 1 [10^−3^ mmHg^−1^] respectively were used. In the aortic model, elastic wrapping resulted in a maximal increased systolic expansion of 176 and 134 mm^2^ for the ‘middle aged’ and ‘elderly’ stiffness properties. As mentioned above, elastic wrapping of the stiff aorta model improved aortic distensibility even if compression forces increased by 5000 Pa/mm (Fig. 3). In contrast, the aorta modeled on a ‘middle-aged’ stiffness profile, demonstrated declining cross-sectional changes for additional forces lower than 3000 Pa/mm. As can be seen in Fig. 3, the normalized improvement in aortic cross section is much lower in the more compliant aortic model.

Overall, these simulation results suggest that elastic wrapping can improve distensibility depending on initial aortic stiffness. A simulation of elastic wrapping using an aortic distensibility of 6 [10^−3^ mmHg^−1^] showed little improvement or even a reduction in distensibility depending on wrap elasticity. Conversely, local distensibility of an aortic model with distensibility of 1 [10^−3^ mmHg^−1^] was increased threefold.

## Discussion

The extra-aortic elastic wrapping surgical procedure has been proposed as a non-pharmacological treatment to improve aortic compliance and reduce pulse pressure and improve coronary perfusion. Our finite element study is the first to use an anisotropic, four-fiber aortic wall model with human tissue material properties to confirm the ability of elastic aortic wrapping to markedly improve distensibility in the stiffened aorta. Furthermore, we present possible limitations regarding the initial aortic distensibility and amount of constriction. Our simulations resulted in the following main findings:

1. The distensibility of a stiffened aorta (distensibility 1 [10^−3^ mmHg^−1^]) when treated with an elastic wrap can be improved from 46 mm^2^ luminal cross-sectional area expansion to 123 mm^2^. Such advances can be made by a 10% constriction relative to diastolic diameter, initial compression of 10000 Pa and a wrapping material with a Young’s modulus of 15 kPa in systole (corresponding to an additional compression force of 1000 Pa/mm) (Fig. 4). The digressive stress-strain response observed in, for example, silicone elastomers are suited for such conditions.
2. More compliant aortas (distensibility 6 [10^−3^ mmHg^−1^]) show almost no improvement upon elastic wrap application. We confirm via our simulations the *in-vivo* results reported by Iliopoulos^7^ and Gillies *et al*.^8^ that show stiff aortas are more likely to be treated effectively with elastic wrapping compared to the young, compliant, native aorta.
3. Our anisotropic constitutive model considering collagen fiber orientation confirmed earlier results of shape distortion upon threshold compression forces^16^. We conclude that to achieve further constriction, the circular aortic cross section will become distorted.

In addition, we hypothesize that the constriction achieved with the elastic wrap can relax otherwise stiffened collagen fibers in the aortic wall, due to overextension, allowing the aortic wall to distend further during a pulsatile systolic pressure rise. An increased distensibility, as predicted by our simulations, is expected to decrease the pulse pressure amplitude. Moreover, we suggest that elastic aortic wrap application will slow wave propagation across the aortic wall, consequently, delaying the return of the secondary pressure wave. Additional studies including fluid-structure-interaction computations and well-designed *in-vivo*/*in-vitro* testing is required to confirm our theoretical models.

Akin to all computer simulations, the results of this study are limited to accurate material test data and mathematical equations appropriately describing natural behavior of the aorta. The four-fiber constitutive model we adopted is one of the best mathematical representations of aortic mechanobiology to date^17,18^. We modeled a single-layered aortic wall best approximated by constituent layers of the aortic wall. We are unaware of any studies that provide detailed stress-strain data of individual aortic layers, e.g. intima, media and adventitia, especially across different age groups. Gao *et al*. report that the stress distribution in a three-layered aortic wall model is more complex than in a single-layered model. On the other hand when comparing a single-layered computational model to a three-layered model, the distribution of simulated velocity, pressure, and areas of deformation are similar^19^. This study was limited to the modelling of an elastic aortic wrap by means of an increased extramural pressure. From a computational perspective, a more precise simulation could include an actual band wrapped around a section of the aorta.

In conclusion, this study presents the first computational simulations of the extra-aortic elastic wrapping procedure, to evaluate possible distensibility improvements in an anisotropic aorta model which employs material data from human subjects. The presented computational models demonstrate a significantly enhanced distensibility in elastically wrapped segments of a stiff aortic wall model, representing aortic stiffening as would be commonly observed in old age. A more compliant, younger aorta model showed no improvement in distensibility under elastic wrapping. Maximal allowable constrictions to avoid aortic shape distortion have been discussed. However, animal *in-vivo* evidence is lacking, and confirmation of the presented computational results is required.

## Methods

A first step to model the effect of elastic wrapping on the aortic compliance was to choose a constitutive equation which accurately describes aortic structural behavior. Our anisotropic simulations account for the determining structural components of the aortic wall, elastin and collagen fibers. There is insufficient data currently to model material properties of the different aortic layers, hence, our model condensed intima, media and adventitia into a single-layer^20^. Using two stiffness models with distensibilities of 6 and 1 [10^−3^ mmHg^−1^] we assess the theoretical potential of the elastic wrap in a compliant and stiff aortic model.

### Constitutive equation

A multitude of different constitutive strain-energy functions have been proposed to describe ascending aorta passive mechanical properties. We adopted an ascending aorta model and parameter fits published by Roccabianca *et al*.^18^, owing to their extensive consideration of available material test data of the aortic wall. Roccabianca *et al*. have previously evaluated biaxial stress-strain data from six published studies and performed a consistent parameter fitting for a phenomenological ‘four-fiber family’ strain energy relation. In this strain energy function (1) the elastin-dominated amorphous matrix is represented by a neo-Hookean relation, whereas a Fung-Type exponential term describes the anisotropic collagen fibers:

1 $${\rm{W}}=\frac{{\rm{c}}}{2}({{\rm{I}}}_{{\rm{c}}}-3)+\mathop{\sum }\limits_{{\rm{k}}=1}^{4}\,\frac{{{\rm{c}}}_{1}^{{\rm{k}}}}{4{{\rm{c}}}_{2}^{{\rm{k}}}}\{{{\rm{e}}}^{[{{\rm{c}}}_{2}^{{\rm{k}}}{({({{\rm{\lambda }}}^{{\rm{k}}})}^{2}-1)}^{2}]}-1\}$$

2 $${{\rm{\lambda }}}^{{\rm{k}}}=\sqrt{{{\rm{M}}}^{{\rm{k}}}\cdot {{\rm{CM}}}^{{\rm{k}}}}$$

3 $${{\rm{M}}}^{{\rm{k}}}={[0,{\sin {\rm{\alpha }}}_{{\rm{o}}}^{{\rm{k}}},\cos {{\rm{\alpha }}}_{{\rm{o}}}^{{\rm{k}}}]}^{T}$$

where c, ${\,{\rm{c}}}_{1}^{{\rm{k}}}$, ${{\rm{c}}}_{2}^{{\rm{k}}}$ and ${{\rm{\alpha }}}_{{\rm{o}}}^{3,4}$ correspond to best-fit material parameters and ${{\rm{I}}}_{{\rm{c}}}$ is the first strain invariant of the right Cauchy-Green deformation tensor, C. ${{\rm{\lambda }}}^{{\rm{k}}}$ indicates the strain of the k^th^ fiber family with fiber orientation ${{\rm{M}}}^{{\rm{k}}}$ at reference point (x,y,z) = (1,0,0), where z denotes the axial coordinate. Axial and circumferential fiber orientation are fixed to be ${{\rm{\alpha }}}_{{\rm{o}}}^{1}=0^\circ$ and ${\,{\rm{\alpha }}}_{{\rm{o}}}^{2}=90^\circ$, respectively. In addition, ${{\rm{\alpha }}}_{{\rm{o}}}^{3}=\,-\,{{\rm{\alpha }}}_{{\rm{o}}}^{4}$, to ensure symmetry and eliminate twisting due to radial pressure loads. For an in-depth discussion of the merits and limitations of this model we refer to Roccabianca *et al*.^18^. This strain-energy relation presents the underlying hyperelastic material model used in all our simulations.

### Numerical simulation

A straight, hollow cylinder with a length of 400 mm and fixed constraints on both ends was used as simplified aortic cross-sectional model computed in the commercial software-package COMSOL Multiphysics 5.3a (COMSOL AB, Sweden). Pressure-zero configurations were selected to yield age-specific diameters and thicknesses at a diastolic inflation pressure of 80 mmHg^17^. Dimensions and respective constitutive material parameters^18^ are presented in Tables 1 and 2. Material test data from human tissue representing a ‘middle aged’ and ‘elderly’ stiffness were originally published by Haskett *et al*.^21^ (age 30–60) and Martin *et al*.^22^ (age 80–98), respectively. Note that we chose two sets of material parameters associated with studies that were found most reliable by Roccabianca *et al*.^18^. The model was axially elongated by 10% representing the inherent axial prestretch of the aorta, and to ensure numerical stability^23–25^. A quasi-static approach to model pressure loads (80, 120, 150 mmHg) was chosen: interestingly time-dependence is commonly neglected in numerical models of the aorta^18,26^. The geometry was discretized with 3-D 20-node quadratic hexahedral solid elements (quadratic serendipity discretization) that exhibit quadratic displacement behavior and have mixed u-P formulation capabilities for simulating fully-incompressible hyper-elastic materials. A final mesh of 12000 quadratic elements (60 circumferential; 200 axial) showed independence of displacement of <1%. The mesh was independent of a second layer through the wall thickness^27^.

**Table 1:** Luminal diameter D_i_ and wall thickness t at pressure-zero configuration and at inflation pressure 80 mmHg.

Ascending aorta model	Pressure-zero configuration		at 80 mmHg^17^	
	D_i_ [mm]	t [mm]	D_i_ [mm]	t [mm]
‘middle aged’	22	2.8	27.9	2
‘elderly’	30	2.4*	31.9	2.3*

*Cuomo *et al*.^17^ use a wall thickness of 2.9 mm for age 75; to achieve a realistic distensibility value, we reduced this to 2.3 mm.

**Table 2:** Best-fit parameters for selected material test-data for the four-fiber family constitutive model of Roccabianca *et al*.^18^.

	${\boldsymbol{c}}$ [kPa]	${{\boldsymbol{c}}}_{1}^{1}$[kPa]	${{\boldsymbol{c}}}_{2}^{1}$	${{\boldsymbol{c}}}_{1}^{2}$[kPa]	${{\boldsymbol{c}}}_{2}^{2}$	${{\boldsymbol{c}}}_{1}^{3,4}$[kPa]	${{\boldsymbol{c}}}_{2}^{3,4}$	$\pm \,{{\boldsymbol{\alpha }}}_{0}^{3,4}\,$[deg]
**Ascending aorta model**								
‘middle aged’	47.43	35.23	7.65E-06	40.84	0.10	15.21	2.58	48.98
‘elderly’	1.56E-08	113.18	17.38	110.27	16.86	121.58	50.17	44.82
**Descending aorta model**								
Garcia Herrera (age 20–35)^30^	37.20	4.68E-06	2.97E-07	2.90E-05	1.68E-05	28.16	3.48	43.88

### Model validation

**Figure 5:**
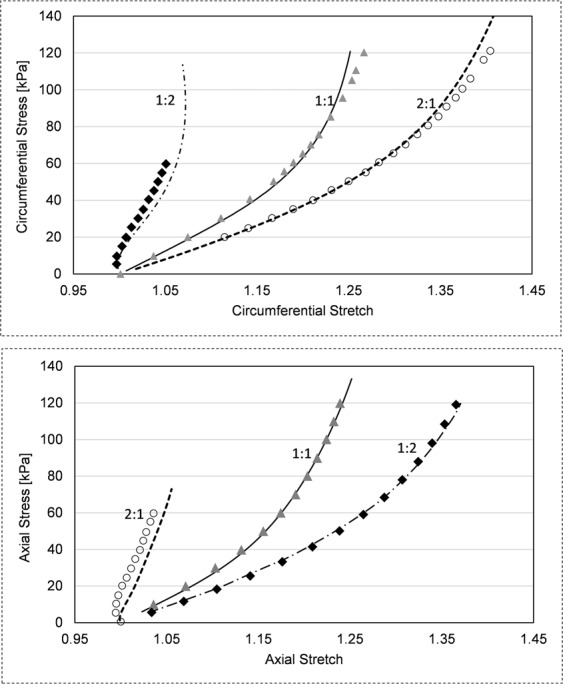
Model validation via biaxial Cauchy stress–stretch data evaluated for three different loading protocols (ratio circumferential to axial stress). Markers represent results reported in Roccabianca *et al*.^18^. Solid lines were obtained by inflation tests of aortic wall model employing the identical constitutive equation and concomitant best-fit parameters.

To validate our finite element analysis, we used our 3D-aortic wall model to reproduce published simulation results according to the boundary conditions of respective studies. Our model using COMSOL Multiphysics 5.3a was able to replicate results published by Roccabianca *et al*.^18^, Baek *et al*.^28^ and de Gelidi *et al*.^29^. Markers in Fig. 5 represent data reported in Roccabianca’s study^18^. The authors simulated biaxial loading protocols of the descending thoracic aorta (material test data by García-Herrera *et al*.^30^ with circumferential to axial stress ratio of 1:2, 1:1 and 2:1. Reproduced values using our aortic wall model are plotted as solid lines (Fig. 5), showing averages of circumferential and axial Cauchy stresses across representative central cut planes. Our validation provided good overall correlation with slight deviations for minimal aortic wall strains.

### Elastic aortic wrap

To describe how the extra-aortic elastic wrapping will be applied, we simulated the compression of an axially-central segment of the simulated aortic wall with forces representing reasonable elastic wrap material behavior. Figure 4(a) illustrates the ascending aorta as preferred location for the elastic wrap placement. The model boundary conditions are schematically depicted in Fig. 4(b). Initially our model aorta was inflated to a diastolic pressure of 80 mmHg. A 40 mm wide central section was then compressed and reduced in diameter up to 37%. Compression forces were varied from 0 to 14000 Pa, adding an additional 1000 to 5000 Pa per millimeter of expansion (Young’s moduli 15000–75000 Pa) to emulate increasing compression during expansion in systole, which represents the stress response in a linear elastic wrapping material. The approximately linear modulus of elasticity at strains greater than 100% (due to the initial wrapping) of potential wrap materials, such as silicone elastomers, can be as low as 2500 Pa^31^.

### Grants

This research was funded by an NHMRC Development Grant. Christian Legerer was supported by an Australian Government Research Training Program (RTP) Stipend and RTP Fee-Offset Scholarship through Federation University Australia.

## Data Availability

The datasets generated during and/or analysed during the current study are available from the corresponding author on reasonable request.
